# Editorial for the Special Issue “Staphylococcal Biology and Pathogenesis”

**DOI:** 10.3390/antibiotics15040398

**Published:** 2026-04-15

**Authors:** Richard A. Proctor

**Affiliations:** School of Medicine and Public Health, University of Wisconsin, Madison, WI 53726, USA; rap@wisc.edu

This Special Issue of *Antibiotics* contains a series of articles that discuss the best approaches to the study of the pathogenesis, immune response, and therapeutics of *Staphylococcus aureus* infections. Over the past decade, we have learned that the immune response to *S. aureus* is extremely complex [1,2,3], at least in part due to the fact that this organism has learned to live within our nares. Several of the most antibiotic-resistant pathogens are grouped as ESKAPE organisms (*Enterococcus* sp. *S. aureus*, *Klebsiella pneumoniae*, *Acinetobacter baumannii*, *Pseudomonas aeruginosa*, and *Enterobacteria* sp.) [4]. These are organisms that live persistently within humans. Sometimes these organisms are called commensals, but this is an inappropriate term in that they are also the organisms that attack humans [5]; a more appropriate term, therefore, is pathobionts. Their persistent colonization suggests that these organisms have evolved to evade the human immune response [1,2,3]. Not only are bacterial mutations responsible for serious and difficult-to-treat infections, but some patients who develop severe and recurrent infections are found to have genetic propensity to these infections [6,7,8] or antibody responses that are non-protective [9], (Contribution 5) or immune-suppressive, e.g., anti-IL6 antibodies [10], high anti-inflammatory cytokine levels, or high IL-10 response to infection [11]. Another line of inquiry has revealed that *S. aureus* carries out a complex interaction with the host based upon metabolic interactions; this is termed immunometabolism, wherein both the host and bacterial metabolism impact the immune response [1,2,3]. Thus, the incredible complexity of these bacterial–host interactions has defined many of the reasons why developing preventive (e.g., vaccines) and therapeutic options (e.g., antibiotics) for *S. aureus* infections has proven to be so difficult to achieve. In this Special Issue, some further details of these interactions are considered.

Central to the study of bacterial pathogenesis and antibiotic susceptibility testing are controlled growth conditions. On the surface, this seems mundane, but the proper use of spectroscopy and flask conditions become critical when attempting to control for the density and growth phase of organisms as well as the availability of oxygen. Each of these parameters has been shown to have a major impact on antibiotic action and the production of multiple bacterial pathogenic and immune evasion factors. Of course, these ultimately determine the pathogenic potential of organisms and their responses to antibiotics. The Somerville laboratory has provided us with details on spectroscopy, which is used to determine the bacterial density and phase of growth (Contribution 1). Previous studies from this lab have shown a flask-to-volume ratio that is important for obtaining reproducible levels of dissolved oxygen in growth media [12]. Hence, the manuscript by Montesinos-Cruz and Somerville provides direction on how to achieve reproducible growth results that can be used in experiments to understand the fundamental questions around bacterial infections.

In a similar vein are the observations of Ersoy et al. (Contribution 2), wherein a common component of tissue culture medium, bicarbonate, is found to influence the susceptibility of bacteria, such as MRSA, to beta-lactam antibiotics. Of course, not only does this have important implications for susceptibility testing, but also has relevance to studies where bacteria are inoculated into tissue culture to study bacterial interactions with host cells. From this work, it is clear that bicarbonate has a major role in defining the activity of antibiotics, not only during susceptibility testing and in tissue culture models of pathogenesis, but also in animal infections.

A major impediment to defining the efficacy of vaccines and antibiotics against *S. aureus* is the lack of a small animal model that replicates the pathogenesis and immune response of humans. It has been discovered that murine models, which have been used widely to define immune responses and the efficacy of antibiotics to many bacterial pathogenic species, do not function well for *S. aureus* because, e.g., mice lack receptors for staphylococcal toxins and show a very different immune response than humans. To address the deficiencies of conventional mouse models, multiple humanized mouse models have been developed to attempt to recapitulate the human immune system in this small animal model. McDonald et al. discuss the development of models in immunodeficient mice that enable the engraftment of human tissue; the authors also discuss the engraftment methods currently used in the field (Contribution 3). They highlight how humanized mouse models successfully uncovered critical human immune responses to various bacterial infections, including *Salmonella enterica serovar Typhi*, *Mycobacterium tuberculosis*, and *Staphylococcus aureus*.

Jura et al. report on the prevalence of immune evasion proteins produced by *S. aureus* in the nose and from wounds in patients with skin infections (Contribution 4). The study found that organisms causing human skin infections contained a diverse repertoire of genes that allow the organisms to evade the human immune system. Perhaps the most significant finding of this study was the prevalence of Ecb, which allows *S. aureus* to suppress the host immune response associated with neutrophil activity.

Further studies on how *S. aureus* evades the host immune system were reviewed by Hajam and Liu (Contribution 5). They discuss the reasons for the failure of all staphylococcal vaccines tested in clinical trials. They propose that the failure of *S. aureus* vaccines is intricately linked to prior host exposure to *S. aureus* early in life, as well as to the pathogen’s capacity to evade adaptive immune defenses, wherein non-protective immune imprints created by previous exposure to *S. aureus* are preferentially recalled by *S. aureus* vaccines. Moreover, they report that IL-10 induced by *S. aureus* plays a unique role in shaping these non-protective anti-staphylococcal immune responses. This finding clearly has important implications for the development of successful staphylococcal vaccines.
